# Fever of Unknown Origin, a Vascular Event, and Immunosuppression in Tick-Endemic Areas: Think About Neoehrlichiosis

**DOI:** 10.7759/cureus.40617

**Published:** 2023-06-19

**Authors:** Cristina Margini, Rafaela Maldonado, Peter Keller, Yara Banz, Robert Escher, Gabriel Waldegg

**Affiliations:** 1 Internal Medicine, Emmental Hospital, Burgdorf, CHE; 2 Infectious Disease, University of Bern, Bern, CHE; 3 Pathology, University of Bern, Bern, CHE

**Keywords:** rituximab, hemophagocytosis, liver biopsy, fever of unknown origin, candidatus neoehrlichia mikurensis

## Abstract

Three patients were referred to our hospital because of fever of unknown origin (FUO) and thrombosis or thrombophlebitis. All of them had been under immunosuppression (IS) with rituximab. Intensive diagnostics for FUO and blood cultures remained negative. Finally, the association of fever, immunosuppression, and a vascular event led to the suspicion of *Candidatus Neoehrlichia mikurensis* (CNM) infection. The diagnosis was confirmed by species-specific polymerase chain reaction (PCR) in the peripheral blood. Therapy with doxycycline or rifampicin led to the resolution of the disease. A liver biopsy was performed in one patient due to hepatomegaly and elevated liver enzymes demonstrating hemophagocytosis. To our knowledge, this is the first histopathological study of liver tissue in CNM infection. The evidence of hemophagocytosis raises the question of whether symptomatic CNM infection might be in part related to host inflammatory and immune responses.

## Introduction

Over the past decade, symptomatic and asymptomatic infections with the tick-borne pathogen *Candidatus Neoehrlichia mikurensis* (CNM) have been increasingly reported [1-20]. CNM was first discovered in 2004 in ticks and wild rodents on the Japanese island of Mikura [21] and first identified as a human pathogen in 2010 in a Swedish patient [1]. This bacterium is part of the family Anaplasmataceae, a small (0.5-1.5 µm), gram-negative, pleomorphic coccus of the intracellular class of alpha-proteobacteria. The bacterium is transmitted by arthropods with a reservoir in rodents [22]. The prevalence of *Ixodes ricinus* ticks ranges from 1% to 22% across the European continent [23]. The first isolation and propagation of CNM in tick cell lines and human primary endothelial cell lines in vitro were achieved in 2021, and a tropism for vascular endothelium as a target cell was demonstrated [14]. This novel emerging disease typically features systemic infection with fever, diffuse myalgia and arthralgia, skin manifestations, and thromboembolic or vascular events. Recently, it has also been demonstrated that asymptomatic immunocompetent individuals can also be infected with this new pathogen [24]. At present, bacterial broad-range or species-specific polymerase chain reaction (PCR) of blood samples has been the only available diagnostic tool, and it is highly likely that many cases of neoehrlichiosis have been missed [22]. With the complete genome (National Center for Biotechnology Information {NCBI} accession number NZ_CP054597) now available, the production of recombinant antigens and the development of serological assays should be possible. The increased awareness of this novel pathogen will reduce the number of undiagnosed and untreated cases. We describe three patients with fever of unknown origin (FUO) and a vascular event, who were ultimately diagnosed with neoehrlichiosis and were treated successfully. We also describe the first histopathological study of the liver in a patient affected by neoehrlichiosis. Two of the cases were previously presented as meeting posters at the annual internal medicine meeting of Schweizerische Gesellschaft für Allgemeine Innere Medizin (SGAIM) Frühjahrskongress on June 3, 2022, at Lausanne (Switzerland).

## Case presentation

Case 1

**Figure 1:**
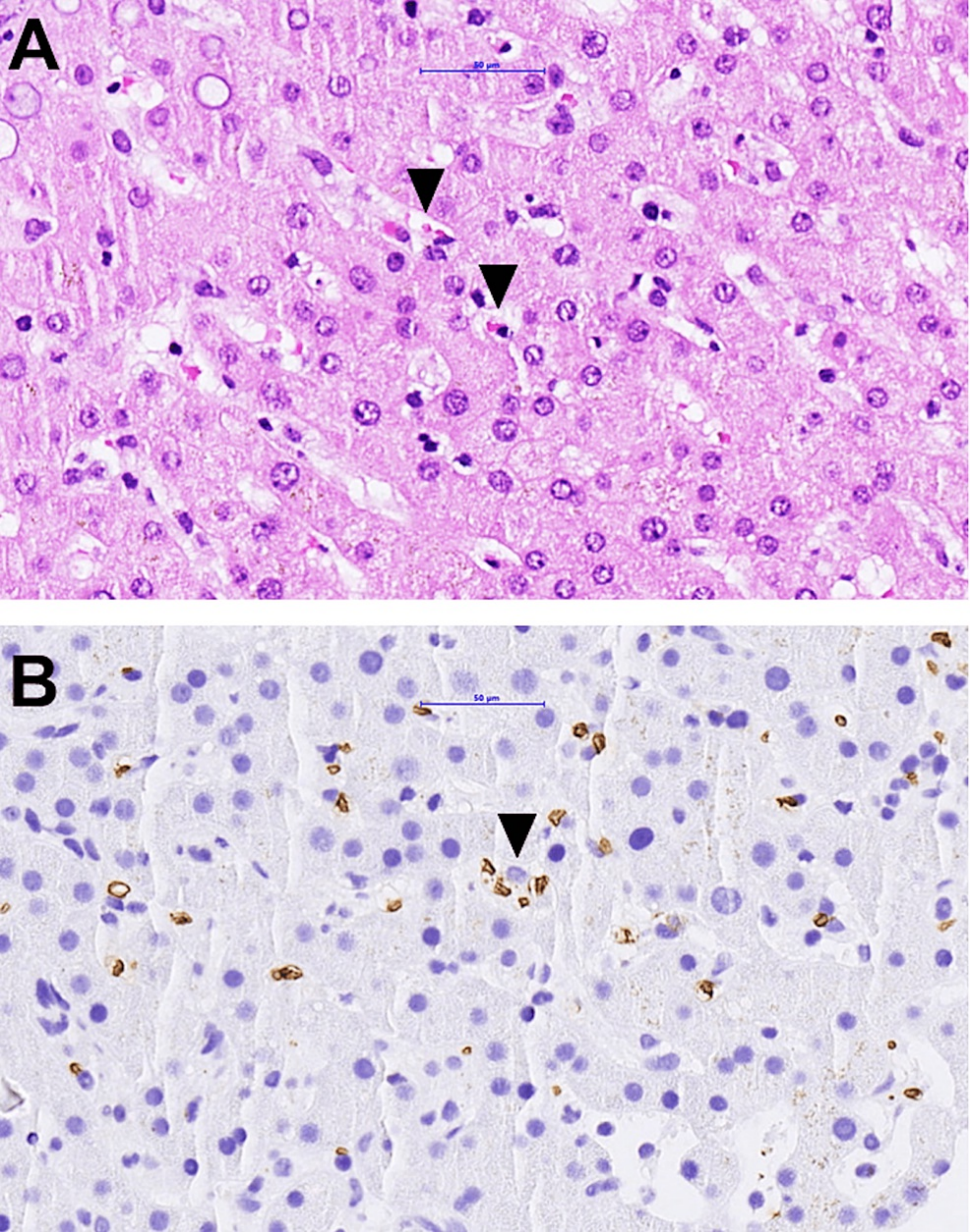
Liver biopsy, 40× magnification, stained with hematoxylin and eosin (panel A) and immunohistochemical staining for glucose transporter 1 (GLUT1) (panel B, brown stain denotes positive reaction in erythrocytes) The arrowheads point to erythrocytes and fragmented erythrocytes undergoing phagocytosis/hemophagocytosis within the body of macrophages in the Disse space

A 66-year-old female was admitted with a four-week history of recurrent fever, chills, myalgia, and weight loss. She had a medical history of type 1 diabetes mellitus, coronary and hypertensive cardiopathy, and erosive destructive rheumatoid arthritis (RA). A history of COVID-19 two months prior without sequelae was reported. The patient had been under maintenance treatment with rituximab every six months and leflunomide daily for four and ten years, respectively. The patient’s medical history was unremarkable with regard to travel abroad, contact with animals other than her own two cats, tick exposure, observed tick bites, or high-risk sexual behavior. The patient was in a reduced general condition, febrile up to 39°C, and suffering from severe chills. Her blood pressure (126/81 mm Hg), pulse (94 beats/minute), and respiratory rate (15 breaths/minute) were within the normal range. On clinical examination, a tender red area measuring 5 cm on the right proximal shin was noted, and sonography confirmed the diagnosis of isolated thrombophlebitis. No clinical signs of active articular inflammation were found. Laboratory parameters were as follows: hemoglobin of 85 g/L (118-158 g/L), C-reactive protein (CRP) of 60 mg/L (0.0-5.0 mg/L), aspartate aminotransferase (ASAT) of 74 U/L (0-35 U/L), alkaline phosphatase of 683 U/L (35-105 U/L), and gamma-glutamyl transferase (GT) of 303 U/L (<40 U/L). Repeated blood cultures remained sterile. Transthoracic echocardiography and computed tomography of the chest and head were unremarkable. Abdominal MRI showed a nonspecific thickening of the jejunal wall, and a hepatomegaly with a craniocaudal diameter of 18.5 cm was found. Fever and chills persisted during follow-up. Leflunomide was discontinued because of a suspected drug fever. Microbiological analyses for SARS-CoV-2; cytomegalovirus; Epstein-Barr virus; hepatitis A, B, C, and E viruses; *Brucella*, *Coxiella*, *Francisella*, *Toxoplasma*, and *Treponema* were negative, and no autoimmune cause was found. A liver biopsy was performed due to hepatomegaly, significantly elevated ferritin of 1322 mg/L (13-150 mg/L), and lactate dehydrogenase of 371 U/L (0-250 U/L). Histopathological analysis revealed evidence of hemophagocytosis (Figure 1).

The triad of FUO, vascular involvement with thrombophlebitis, and immunosuppression (IS) led to the suspicion of neoehrlichiosis, despite the absence of tick bites. Finally, the diagnosis was confirmed by positive PCR for CNM DNA in the peripheral blood. The patient was treated orally with doxycycline 100 mg twice daily. Doxycycline was switched to rifampicin 300 mg twice daily because of the severe side effects of dizziness. Within a few days, the fever subsided, and laboratory findings normalized. The patient recovered completely with a negative PCR for CNM four weeks after treatment initiation. Anticoagulation for thrombophlebitis was continued for three months.

Case 2

A 59-year-old male was referred to our hospital with FUO. Ten years earlier, a splenectomy led to the diagnosis of hairy cell leukemia, which was treated with several cycles of rituximab-containing chemotherapy. The patient reported a one-month history of recurrent twice-daily fevers prior to admission. Although the patient frequently walked and hiked in the woods, no tick bites were reported. On admission, the patient was febrile (38.1°C). Laboratory analysis showed a leukocyte count of 16×10^9^/L (3.6-10.5×10^9^/L), a CRP of 156 mg/L (0.0-5.0 mg/L), and slightly elevated alanine aminotransferase (ALAT) of 95 U/L (0-35 U/L) and gamma-GT of 128 U/L (<40 U/L). The serological tests for cytomegalovirus, Epstein-Barr virus, *Brucella*, *Toxoplasma*, and HIV were negative. Repeated blood cultures remained sterile. A computed tomography scan of the chest, abdomen, and pelvis did not reveal the cause of the fever, and there were no signs of an inflammatory or infectious process. A recurrence of the hairy cell leukemia was excluded through bone marrow biopsy. During hospitalization, the patient developed a deep vein thrombosis of the left leg, involving the common femoral and popliteal vein. Therapeutic anticoagulation with rivaroxaban was started. The triad of FUO, deep vein thrombosis, and immunosuppression led to the suspicion of neoehrlichiosis. PCR analysis for CNM in the peripheral blood was positive. Doxycycline 100 mg twice a day was started for six weeks. Within days, the patient was afebrile. The PCR test was negative at the end of treatment and remained so at the one-month follow-up. After three weeks of therapy with rivaroxaban, anticoagulation was switched to a vitamin K antagonist because of persistent venous stasis symptoms. One year after the diagnosis, an endovenectomy of the left common femoral vein and left superficial femoral vein was successfully performed [25].

Case 3

**Figure 2:**
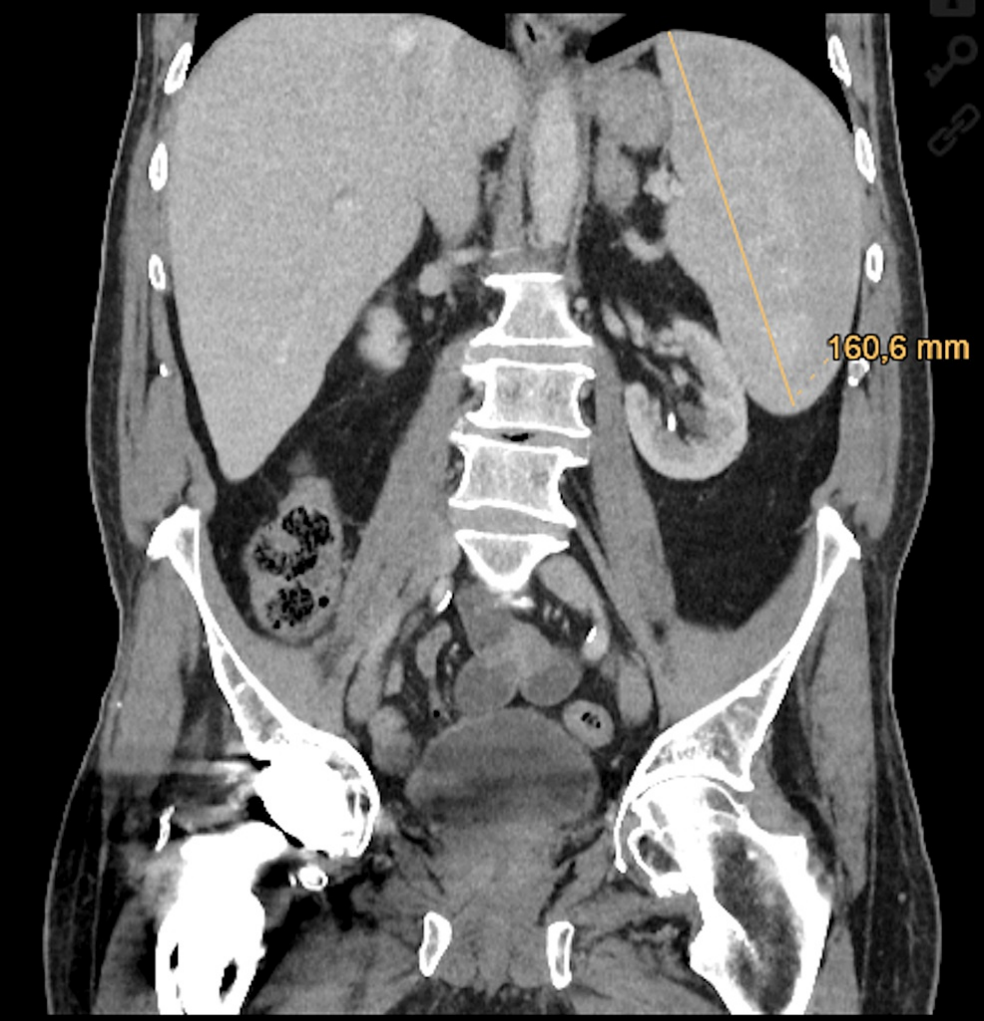
Computed tomography of the chest and abdomen with splenomegaly

A 73-year-old male was referred to our hospital with FUO. He had a history of mantle cell lymphoma treated five years before using a rituximab-based regimen, secondary immunoglobulin deficiency, and coronary artery disease. The patient presented to our hospital with six months of recurrent fever of up to 39°C, fatigue, and stress intolerance. Four months prior to presentation, a bilateral thrombosis of the cephalic veins was diagnosed and treated with rivaroxaban. As a former farmer living in a rural area, he could recall several tick bites in the past. On presentation, laboratory tests showed a hemoglobin of 119 g/L (125-172 g/L), leukocyte count of 4.9×10^9^/L (3.6-10.5×10^9^/L), platelets of 104×10^9^/L (160-370×10^9^/L), CRP of 60 mg/L (0.0-5.0 mg/L), and normal liver enzymes. The serological tests for cytomegalovirus and Epstein-Barr virus were consistent with a past infection and were negative for *Treponema*, *Toxoplasma*, *Leptospira*, *Brucella*, and *Francisella*. The computed tomography of the chest and abdomen revealed splenomegaly without lymphadenopathy (Figure 2).

The triad of FUO, bilateral arm vein thrombosis, and immunosuppression led to the suspicion of CNM infection, which was confirmed by the specific positive PCR in the peripheral blood. Therapy with doxycycline 100 mg twice a day was started for four weeks. Within days, the patient became afebrile, und laboratory findings normalized. Therapeutic anticoagulation with rivaroxaban was continued for a total of six months. The patient’s characteristics are summarized in Table 1.

**Table 1:** Patient’s characteristics

	Underlying disease	Type of immunosuppression	Time from symptom onset to diagnosis	Thrombotic event	History of tick bites
Patient 1	Rheumatoid arthritis	Rituximab and leflunomide	6 weeks	Thrombophlebitis of the right ankle	No
Patient 2	Hairy cell leukemia and splenectomy	Rituximab and chemotherapy	4 weeks	Deep vein thrombosis of the left leg	No
Patient 3	Mantle cell lymphoma and immunoglobulin deficiency	Rituximab and bendamustine	6 months	Bilateral thrombophlebitis of the upper extremities	Yes

## Discussion

In our three case reports, FUO together with a vascular event was the initial clinical presentation of neoehrlichiosis. Laboratory findings are generally unspecific and include leukocytosis, anemia, and elevated CRP [22]. Since CNM does not grow in routine blood cultures, the initial clinical presentation may be misinterpreted as a recurrence of an underlying rheumatologic, hematologic, or neoplastic disease [7]. As a result, diagnosis is often delayed, and the patients may undergo an odyssey of diagnostic procedures before a definitive diagnosis is made, as in our three patients. Tick bites transmit CNM in endemic areas. Less than half of the patients affected by neoehrlichiosis recall a tick bite, making it a poor predictor of a tick-borne disease [22]. Patient 1 may have had indirect contact with ticks through close contact with her cats, and patient 2 was an active outdoorsman with potential contact with arthropods.

The prevalence of CNM in ticks collected in an area around Zurich, Switzerland, was as high as 8% [6], and the estimated prevalence in Europe ranges from 0% to 22% [23]. Thus, CNM infection should also be suspected in the absence of recalled tick bites in endemic areas. In Europe, the tick *Ixodes ricinus* is widespread; it is a vector for *Borrelia burgdorferi*, tick-borne encephalitis virus, *Francisella tularensis*, CNM, and *Anaplasma phagocytophilum*, the pathogen of human granulocytic anaplasmosis (HGA). CNM and *Anaplasma phagocytophilum *belong to the family of Anaplasmataceae, together with the genera *Ehrlichia* and *Neorickettsia* [26]. In contrast to North America, HGA is much less prevalent in Europe, while CNM hast not yet been reported in North America [26]. Both diseases share some clinical similarities such as the transmission via tick bites, the unspecific febrile illness, and inflammatory state with chills, malaise, elevated liver enzymes, and vascular complications [26].

Ticks are frequently coinfected with two or more pathogens; the prevalence of co-infection depends on geography. For example, in Europe, co-infections in *Ixodes ricinus* ticks are as high as 12.5% in studies from Ukraine and significantly less in Norway (3.3%) [27]. In our three cases, co-infection with other tick-borne-associated pathogens was excluded clinically and by serological analysis. A recently published case series from Sweden described vascular complications ranging from deep vein thrombosis to pulmonary embolism, transitory ischemic attacks, and arterial aneurysms as central features of CNM infection rather than fever [16]. Similarly, in our three cases, vascular involvement was a characteristic symptom. It has been postulated that vascular complications in neoehrlichiosis may be related to CNM endotheliotropism. In support of this hypothesis, CNM was recently cultivated in human endothelial cells and detected in circulating endothelial cells of infected individuals, but a direct visualization of CNM in vascular biopsies is so far lacking [16].

Two of our patients presented with slightly elevated liver enzymes. To our knowledge, no histopathological liver features in CNM infection have yet been published. In the liver biopsy of patient 1, we observed the activation of macrophages and the phagocytosis of red blood cells (Figure 1) as evidence of hemophagocytosis. A diffuse activation of the mononuclear phagocyte system, including the activation of recruited and resident hepatic mononuclear phagocytes, has been described in the liver tissue of subjects affected by *Ehrlichia chaffeensis*, another obligate intracellular bacterium belonging to the family Anaplasmataceae, whose genome has a substantial similarity to CNM [28]. Patient 1 presented some criteria pointing toward the diagnosis of hemophagocytic lymphohistiocytosis (HLH): fever, hypertriglyceridemia, hemophagocytosis in liver biopsy, and elevated ferritin. However, soluble cluster of differentiation 25 (CD25) (soluble interleukin 2 (IL-2) receptor alpha) and natural killer cell activity were not tested, and the diagnosis of HLH syndrome could not be confirmed [29]. A case of HLH associated with *Anaplasma phagocytophilum *infection has also been published [30]. Symptomatic CNM infection might be in part related to host inflammatory and immune responses, explaining the clinical picture with prolonged fever, chills, and inflammatory signs. This hypothesis could be supported by the evidence of pro-angiogenic and type 1 cytokine production as a part of the host response in patients with neoehrlichiosis, independent of immunity, and raised levels of cytokines in neoehrlichiosis patients with compromised B cell function [31]. All patients in our study were immunosuppressed with a rituximab-containing regimen, either ongoing or in the past. The available literature suggests that immunosuppression and especially the depletion of the B cell host response are decisive risk factors for symptomatic CNM and disease severity. We performed a literature review in PubMed of all symptomatic cases of CNM infection (keywords: *Candidatus Neoehrlichia mikurensis *and neoehrlichiosis) described until March 2023. We only considered articles with clinical information about the underlying disease and the type of immunosuppression (IS). A total of 20 articles, describing 70 symptomatic CNM cases, were found​​​​​ (Table 2).

**Table 2:** Case report/series of CNM infection Listed are case series with symptomatic patients; not included are case series with asymptomatic subjects only and articles without clinical information about immunosuppression and underlying disease *These articles also described cases already published; this table presented only the original cases presented in each article N, number of patients; IS, immunosuppression; B-CLL, B cell chronic lymphocytic leukemia; CLL, chronic lymphocytic leukemia; SLE, systemic lupus erythematosus; T-LGL, large granular T-lymphocytic leukemia; DLBCL, diffuse large B cell lymphoma; R-CHOP, rituximab, cyclophosphamide, doxorubicin hydrochloride, vincristine sulfate, and prednisone; R-HAM, rituximab, high-dose cytosine arabinoside, and mitoxantrone; Co, corticosteroids; CP, cyclophosphamide; Mtx, methotrexate; Aza, azathioprine; Rtx, rituximab; Ch, chemotherapy ; Inx, infliximab; CNM, *Candidatus Neoehrlichia mikurensis*

Author and year	Country	N	IS (n)	Underlying disease (n)	IS type (n)
Welinder-Olsson et al., 2010 [1]	Sweden	1	Yes	CLL and splenectomy (1)	Co and CP (1)
von Loewenich et al., 2010 [2]	Germany	2	Yes (1)	Chronic inflammatory demyelinating polyneuropathy	Rtx, Co, and CP (1)
Fehr et al., 2010 [3]	Switzerland	1	No	Coronary artery bypass	No
Pekova et al., 2011 [4]	Czech Republic	2	Yes	Mantle cell lymphoma and splenectomy (1) and posttransplant lymphoproliferative disorder (1)	R-HAM, Mtx, and CP (1) and Rtx and tacrolimus (1)
Li et al., 2012 [5]	China	7	No	No	No
Maurer et al., 2013 [6]	Switzerland	2	Yes (1)	History of CLL (1) and follicular lymphoma in remission (1)	Rtx (1)
Grankvist et al., 2014* [7]	Sweden	5	Yes	B-CLL (1), follicular lymphoma and SLE (1), T-LGL and psoriasis arthropathy (1), psoriasis (1), DLBCL and rheumatoid arthritis (1), and splenectomy (4)	CP (1), CP and Co (2), Co (1), and Aza and Co (1)
Grankvist et al., 2015 [8]	Sweden	2	No	No	No
Andréasson et al., 2015 [9]	Sweden	1	Yes	Rheumatoid arthritis	Rtx
Grankvist et al., 2015* [10]	Sweden	3	Yes	Rheumatoid arthritis (1), granulomatosis with polyangiitis (1), and pre-B acute lymphocytic leukemia (1)	Mtx and Rtx (1), Rtx (1), and mercaptopurine, Mtx, and Co (1)
Dadgar et al., 2017 [11]	Sweden	1	Yes	Multiple sclerosis	Rtx
Frivik et al., 2017 [12]	Norway	1	Yes	Cured Hodgkin’s lymphoma and splenectomy (1)	Ch (1)
Wass et al., 2018* [13]	Sweden	8	Yes (6)	Follicular lymphoma (1), B-CLL (1), B-CLL and splenectomy (1), polymyalgia rheumatica (1), primary hypogammaglobulinemia and splenectomy (1), focal segmental glomerulosclerosis (1), autoimmune hemolytic anemia, and Crohn’s disease and splenectomy (1)	Rtx and Ch (1), Rtx and Co (1), ibrutinib and Ch (1), Co (2), and Co and Aza (1)
Waas et al., 2019* [14]	Sweden	4	Yes	Multiple sclerosis (2), granulomatosis with polyangiitis (1), and B-CLL (1)	Rtx (2), Rtx and Ch (1), and Rtx, Ch, and Co (1)
Quarsten et al., 2021 [15]	Norway	12 (eight with symptoms)	Yes (8)	Rheumatoid arthritis (2), psoriatic arthritis (2), sacroiliitis (1), Crohn’s disease (2), and ulcerative colitis (2)	Inx (7) and Rtx (1)
Höper et al., 2021* [16]	Sweden	15	Yes (9/15)	Follicular lymphoma (2), Waldenström macroglobulinemia (1), multiple sclerosis (3), previously healthy (3), metabolic syndrome (2), polymyositis (1), previous Hodgkin’s disease (1), rheumatoid arthritis (1), and atrial fibrillation and aortic aneurysm (1)	Unknown
Lenart et al., 2021 [17]	Slovenia	1	Yes	DLBCL and splenectomy (1)	R-CHOP
Boyer et al., 2021 [18]	France	4	Yes (1)	CLL and splenectomy (1) and follicular lymphoma in remission (1)	Ibrutinib (1)
Sjöwall et al., 2021 [19]	Sweden	1	Yes	Malignant lymphoma and splenectomy (1)	Rtx
Bamford et al., 2023 [20]	South Africa	1	No	No	

Most of the symptomatic patients were immunodeficient because of immunosuppressive therapy (N=45) or splenectomy. Many patients (N=17), similar to the presented cases, had rituximab or rituximab-based chemotherapy, and a considerable number had infliximab (N=7), cyclophosphamide, and/or corticosteroids. In nine patients, the type of immunosuppressive therapy was not specified. It appears that rituximab treatment might be associated with a higher risk of developing symptomatic neoehrlichiosis. Similarly, recent published studies showed higher infection rates in patients with multiple sclerosis [32] and rheumatoid arthritis [33] treated with rituximab. Rituximab targets CD20 on B cells and is related to a depletion of all stages of B cells, producing hypogammaglobulinemia. Anti-CD20-targeted therapies also modulate T cell function [34]. Since T cell function is crucial in the clearance of intracellular pathogens such as CNM, this could explain the vulnerability of patients treated with rituximab. CNM responds to antibiotic treatment, and all our patients recovered promptly within days after starting treatment with doxycycline. In patient 1, treatment was switched to rifampicin because of side effects, confirming its efficacy, as previously reported in another case [3]. There is no consensus on the optimal length of treatment. The average reported treatment duration is three weeks [24]. Patient 1 was treated for two weeks with doxycycline and switched to rifampicin for further two weeks because of side effects. Patient 2 was treated for six weeks, since PCR for CNM was still positive two weeks after the start of treatment, and patient 3 was treated for four weeks. In all three cases, the success of treatment was confirmed clinically and by means of a negative CNM PCR at the end of treatment.

## Conclusions

The diagnosis of CNM infection is challenging. In patients under immunosuppression, especially rituximab, who present with fever and a vascular event, CNM should always be suspected in tick-endemic areas. A diagnostic PCR in peripheral blood must be performed. The demonstration of hemophagocytosis in the liver tissue of one CNM-infected patient might suggest an extensive host inflammatory and immune response in some patients with neoehrlichiosis.
